# A Neglected Profession

**Published:** 1885-07

**Authors:** George E. Blackham


### A NEGLECTED PROFESSION.

GEORGE E. BLACKHAM, M. D.

In these days it is hardly necessary to argue much in favor of the idea that persons entering upon any profession should, before beginning to practice it, be prepared for its responsibilities and difficulties by a special course of education. The statement is, indeed, almost axiomatic, and the reasonableness of the proposition has been so far recognized by law that for some of the professions evidence of such preparatory education is required as the legal perquisite to practice. The old idea of a "liberal education" is not regarded as sufficient. There must be a special education with special reference to the profession.

Medicine, theology and law are thus protected and the institution of State Normal schools for the special education of teachers shows that teaching is coming to be regarded, and very properly so, as one of the learned professions for which special education is necessary. There is, however, one profession more sacred in its functions and more important to the state and the individual than any other, which in the great majority of cases is entered upon not only without special preparation but after a course of education which not only fails to give the necessary knowledge, but absolutely tends to unfit those upon whom the sacred and important functions of this profession devolve for their proper exercise. I refer to the profession of motherhood.

What calling can be more sacred and important than that of the woman who brings into the world a child, and upon whose knowledge, tact and skill depend the direction in which it is started mentally, morally and physically upon its journey through life? And yet, how large a proportion of those who assume this sacred and awful responsibility do so not only in total ignorance of its dangers and its duties, but unfitted mentally and physically by the strain of acquiring useless accomplishments, for avoiding its dangers and performing its duties even if they were not ignorant of them.

The eminent Dr. Thos. A. Emmet, long surgeon-in-chief of the Woman's Hospital, New York, says in his recent work on Gynæcology—"On looking over my case books, I have been surprised to find the same statement repeated again and again that the sufferer had taken the highest honors at some girls' school or college." And this, I think is, in the main, the experience of all physicians. Girls who have, during the period of devolopment, diverted the nervous force required for development of the physical organization to the over-development of the brain and nervous system are almost sure to become sufferers thereby, and not only that but to transmit the evil effects to their children, if they have still vitality enough left to bear children. And for what is this sacrifice made? For useful knowledge, for knowledge that will enable them to bring forth, to bear, to train children blessed with sound minds and sound bodies, children that shall be an honor to them mentally, morally, socially and physically? Not at all. The majority of these young women leave college densely, yes criminally, ignorant of almost everything a woman ought to know before assuming the duties and responsibilities of wifehood and motherhood.

There seems to be an idea prevalent that young women should be left in ignorance of these things because the discussion of them would be indelicate and tend to take away that fine bloom of modesty which is one of the chiefest charms of girlhood. This is simply rank nonsense. The girl passing from childhood to young womanhood can not be unconscious of the change mental, moral and physical, of the development of new hopes, new fears, new ideals, and new physical conditions. Better, far better, for her and for those to come after her, that wise and kindly instructions should point out the true meaning of these changes, of the new responsibilities that come with them, and the new dangers to be avoided. Better, morally as well as physically, that the intellect be instructed than that the imagination be left to brood unaided, undirected, and at last, perchance, to become morbid.

Of course much of the instruction should be given at home and by mothers; but too often the mothers themselves do not know, and much of the instruction, if given at all, must be given in school. This, of course, precludes any idea of the co-education of boys and girls after the age of ten or twelve years, but this so far from being a thing to be regretted would in itself be a most valuable improvement on our present method, which I believe to be demoralizing in the extreme.

It may be asked, would I forbid to women the higher studies, classics, mathematics, music, painting, etc., etc. No; but I would first develop their bodies so that they might be sound and fitting caskets for the sound minds within; next I would store their minds with useful knowledge specially adapted to the role that most of them must, and any of them may, be called upon to play in life, and after that, if time and circumstances permitted, these other things, in moderation, might be added unto them.

There *may* be women preachers, and doctors and lawyers and what not, but, if the race is to be perpetuated, there *must* be mothers, and they must be in the majority; hence, on the doctrine of "the greatest good of the greatest number," our educational scheme for girls should be such as to prepare them for their great and sacred calling, and even to the others this training would not be wasted for the most advanced of the women emancipators are liable to lapse into matrimony though, happily, not nearly so likely to be promoted to the dignity of motherhood.

Suppose we should come to regard motherhood in fact, as most of us already do in sentiment, as not only the most sacred of relations but the most honorable and dignified of professions, that our schools should aim to prepare our girls for its dignities, its duties, and its trials by training their bodies, developing their minds, teaching them the laws which the Creator has indelibly engraven upon their organizations and which are as much His laws as are those brought down by the law giver of Israel from the heights of Sinai. Suppose no girl could *graduate* till she new how to take proper care of her own health and had some idea as to the feeding, dressing and training of young children, would not the prospect for the health, happiness and virtue of future generations be brighter, would there not be fewer suffering mothers and sickly children, fewer unhappy marriages and fewer divorces; and, last but not least, would it not tend to diminish the number of those wives who, driven by suffering, counseled by ignorance, strive to avoid the pangs and duties and glories of motherhood and thereby earn unto themselves more certain damnation in this world in the shape of broken health and physical suffering and in the hereafter only the prospect of standing under the banner of that Tetrarch of Galilee who is known to history chiefly for his "slaughter of the innocents?"

Is it not time that false modesty gave way to true, that motherhood should be regarded as a noble profession to be prepared for as gladly, as proudly, and as thoroughly as law, medicine or theology? Is it not time that we ceased to educate girls on the principle of the man who said "Give me the luxuries of life and I will dispense with the necessities?"

